# Detection of classical BSE prions in asymptomatic cows after inoculation with atypical/Nor98 scrapie

**DOI:** 10.1186/s13567-023-01225-2

**Published:** 2023-10-04

**Authors:** Marina Betancor, Belén Marín, Alicia Otero, Carlos Hedman, Antonio Romero, Tomás Barrio, Eloisa Sevilla, Jean-Yves Douet, Alvina Huor, Juan José Badiola, Olivier Andréoletti, Rosa Bolea

**Affiliations:** 1 Centro de Encefalopatías y Enfermedades Transmisibles Emergentes, Facultad de Veterinaria, Instituto Agroalimentario de Aragón - IA2, Universidad de Zaragoza, 50013 Zaragoza, Spain; 2 Servicio de Cirugía y Medicina Equina, Hospital Veterinario, Universidad de Zaragoza, 50013 Zaragoza, Spain; 3 UMR INRAE ENVT 1225 Interactions Hôtes-Agents Pathogènes, École Nationale Vétérinaire de Toulouse, 31076 Toulouse, France

**Keywords:** Prion, PrP^Sc^, atypical scrapie, bovine spongiform encephalopathy

## Abstract

The emergence of bovine spongiform encephalopathy (BSE) prions from atypical scrapie has been recently observed upon experimental transmission to rodent and swine models. This study aimed to assess whether the inoculation of atypical scrapie could induce BSE-like disease in cattle. Four calves were intracerebrally challenged with atypical scrapie. Animals were euthanized without clinical signs of prion disease and tested negative for PrP^Sc^ accumulation by immunohistochemistry and western blotting. However, an emergence of BSE-like prion seeding activity was detected during in vitro propagation of brain samples from the inoculated animals. These findings suggest that atypical scrapie may represent a potential source of BSE infection in cattle.

## Introduction, methods and results

Transmissible spongiform encephalopathies, or prion diseases are fatal neurodegenerative disorders produced by the accumulation of a neurotoxic misfolded isoform (PrP^Sc^) of the cellular prion protein (PrP^C^). This conformational change provides PrP^Sc^ with partial resistance to proteases and a tendency to form aggregates, characteristics that have been extensively used for the diagnosis of these disorders. The presence of the pathogenic protein in a host can be due to three possible origins: sporadic or idiopathic, familial (caused by the mutation of the *PRNP* gene, encoding PrP^C^) or acquired through exposure to prions. Prion diseases in animals include scrapie in small ruminants, chronic wasting disease (CWD) in cervids and bovine spongiform encephalopathy (BSE) in cattle. The classical form of BSE (C-BSE) caused one of the most important food safety crises in history, due to its link to the variant form of Creutzfeldt-Jakob disease (vCJD) in humans [1].

Among animal prion diseases, atypical scrapie in sheep and goats has the widest geographical distribution [2, 3]. First described in 1998 in Norway [4], this atypical form presents significant neuropathological, clinical, biochemical and epidemiological differences compared to classical scrapie. The origin of atypical scrapie has been widely discussed. Due to its epidemiological characteristics, it has been suggested that atypical scrapie is a spontaneous prion disease [5]. Results obtained in murine models of spontaneous scrapie seem to indicate that this is indeed the case [6]. In 2019, the emergence of C-BSE from atypical scrapie was demonstrated. The transmission of atypical scrapie isolates to bovine PrP transgenic mice led to the propagation of C-BSE prions. The presence of low levels of the C-BSE agent was also confirmed in the original ovine isolates by protein misfolding cyclic amplification (PMCA) [7], an ultrasensitive technique for the detection of prions. The emergence of C-BSE after interspecies transmission of atypical scrapie was also observed by us in a different host: pigs inoculated with atypical scrapie. Although intracerebral inoculation of the agent did not cause disease in the pigs, C-BSE prions were detected in several brain areas of these animals using PMCA, suggesting that pigs could act as a reservoir for BSE [8].

Due to this link between atypical scrapie and the emergence of BSE, the objective of this study was to determine whether the inoculation of an atypical scrapie isolate could induce BSE-like disease in cattle. In this report, we describe for the first time the emergence of C-BSE-like prions during the in vitro propagation of brain samples from calves inoculated with an atypical scrapie isolate.

Four 10-month-old Pyrenean breed calves were used. Animals were intracerebrally challenged with 1 mL (10% brain homogenate w/v in sterile saline solution) of the PS152 isolate. This isolate was made from the brain of an AFRQ/AFRQ (amino acid at codons 136, 141, 154 and 171 of the *PRNP* gene) clinical sheep naturally affected by atypical scrapie, and provided by UMR INRAE ENVT 1225, Interactions Hôtes Agents Pathogènes, École Nationale Vétérinaire de Toulouse. Calves were placed under general anesthesia and the trephine was done using a dental drill positioned paramedially in the mid-frontal region of the head, at an angle of 90° with respect to the rostro-caudal slope of the front of the skull. The inoculum was injected into the frontal cortex via a 9-cm-long needle (gauge, 22).

Cows were housed together in the BSL3 facility from Centro de Encefalopatías y Enfermedades Transmisibles Emergentes of the University of Zaragoza, and daily monitored by animal husbandry staff. Veterinary clinical assessments were performed monthly in order to detect any clinical sign compatible with a prion disease. Animals were euthanized by intravenous pentobarbital injection followed by exsanguination without showing clinical signs of prion disease. Post-inoculation periods and reasons for euthanasia are shown in Table 1. Bovine 1 was euthanized due to the compromise of its welfare after the development of concomitant pathologies. The other animals were selectively euthanized to test the evolution of the disease.

**Table 1:** **Atypical scrapie-challenged cows**

ID	Isolate	Sex	Years post-inoculation	Reason for euthanasia
Bovine 1	PS152	Female	10.1	Septic footpad, laminitis
Bovine 2	PS152	Female	7.2	Elective
Bovine 3	PS152	Female	11.3	Elective
Bovine 4	PS152	Female	11.3	Elective

Animals were sacrificed without showing clinical signs of TSE.

Necropsies were conducted systematically, and tissue samples were collected from the central nervous system (CNS) using sterile prion-free material and equipment. All samples were collected in duplicate, one sample was fixed in 10% formalin for the histological analysis and the other sample was stored at −80 °C for further biochemical and in vitro assays.

Formalin-fixed tissues were processed according to standard histopathological procedures. Sections were stained with hematoxylin and eosin for the histopathological study. PrP^Sc^ detection in brain sections was performed by immunohistochemistry using the monoclonal primary antibodies L42 and 6H4 (Biopharm, Darmstadt, Germany), as described previously [9, 10].

Samples from frontal cortex (Fc), thalamus (Th), cerebellum (Cbl) and obex from the four inoculated cows and two negative age-paired controls (11 and 12 years old) were subjected to western blotting for PrP^Sc^ detection. 20 µL of sample was mixed with 180 µL of 10% negative brain sheep homogenate, as previously described [11]. Then, the TeSeE Western blot kit (Bio-Rad, France) was used following the manufacturer’s recommendations to perform the PrP^Sc^ extraction and samples were subjected to a 12% SDS/PAGE and transferred to PVDF membranes. After blocking for 30 min in PBST containing 2% BSA, PrP^Sc^ immunodetection was performed with the monoclonal antibodies 12B2 (mAb 1:10 000, Creative Biolabs’) and Sha31 (mAb, 1:8000, SPI-Bio), a secondary antibody HRP-conjugated anti-mouse (1:5000; Bio-rad) and ECL substrate (Pierce) to reveal peroxidase activity.

Homogenates (10% in saline solution) were prepared using tissue from the previously mentioned brain areas and animals. Each homogenate was later diluted 1/50 to prepare the PMCA seeds. These seeds were then subjected to three rounds (24 h each) of PMCA, as previously described [11]. 5 µL of seed and 45 µL of substrate were mixed per well in a 96-well PCR microplate (Axygen Scientific, USA). One Teflon bead (2.381 mm diameter) was added to each well. Amplification was performed in a Qsonica Q700 sonicator, using a water recirculation system. Microplates were then submitted to 96 cycles of 10 s sonication (75% power) followed by a 14 min and 50 s incubation period at 39.5 °C temperature. After the PMCA round (24 h), 5 µL of the reaction product was added to a new microplate containing 45 µL of fresh substrate and a new round with 96 cycles of sonication and incubation was performed. Brains from TgBov mice, overexpressing bovine PrP [12, 13] supplemented with 0.25% final concentration of low molecular weight dextran (Sigma-aldrich, D4911-10G mol wt 6500/10 000) were used as substrate. TgBov mice were selected as the used substrate for PMCA since bovines are the natural hosts of BSE, and therefore this model could correctly mimic BSE prion transmission in natural conditions. Unseeded substrate was used as negative control, and the positive control was performed using cattle C-BSE isolates diluted from a 10^−1^ to a 10^−8^ dilution. PrP^Sc^ detection after PMCA was performed by western blot. Prior to western blot, a BCA (bicinchoninic acid) protein assay (23227, Thermo Scientific™) was done to determine the total protein concentration of samples and equivalent amount of proteins were loaded in each well. PrP^Sc^ band ratio of the resulting PMCA products and 6 C-BSE controls were determined using the Image J software.

**Figure 1:**
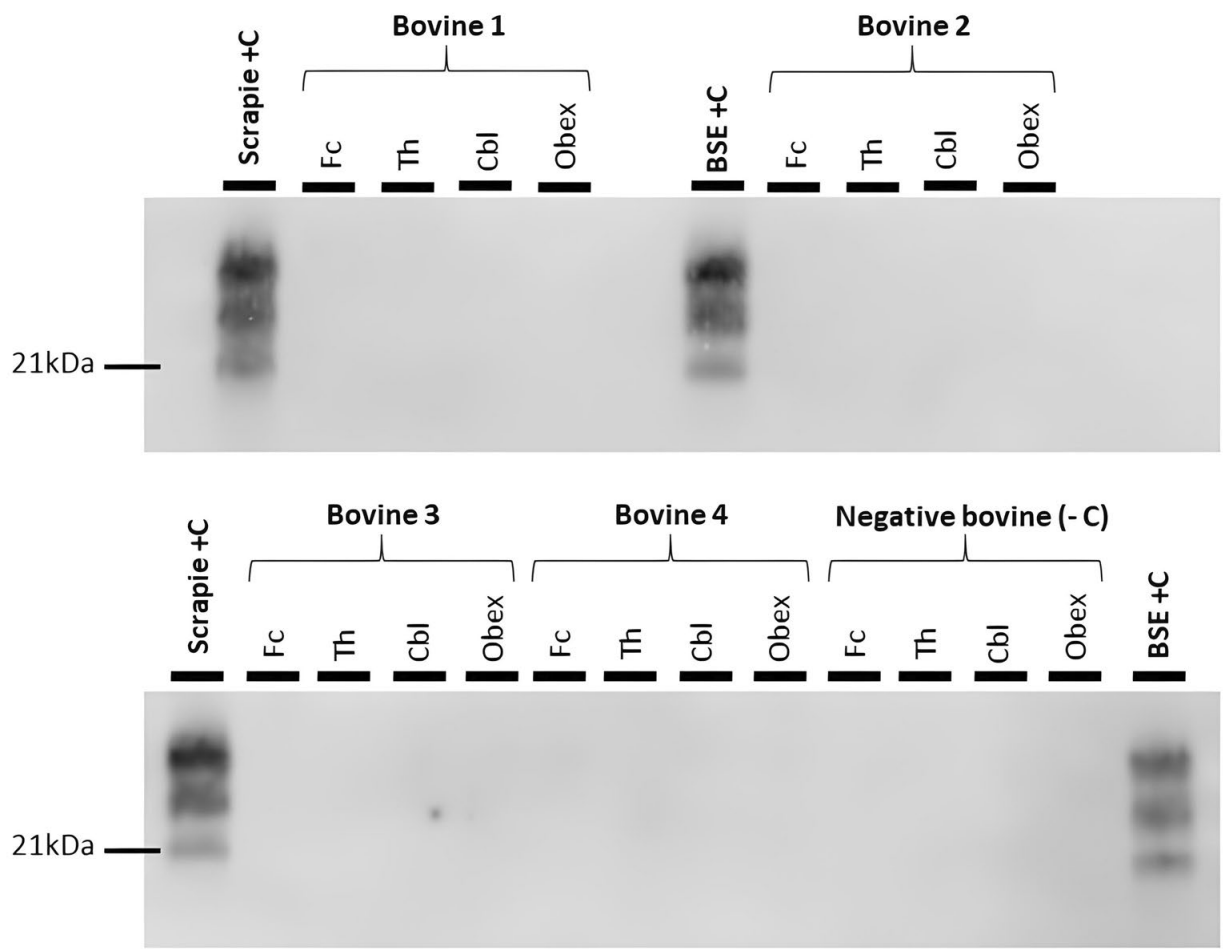
**No PrP**^**Sc**^ **accumulation by western Blot is detected in brains from cows intracerebrally challenged with atypical scrapie**. Frontal cortex (Fc), thalamus (Th), cerebellum (Cbl) and obex homogenates from the four atypical scrapie-challenged cows and a negative control cow were subjected to PrP^Sc^ detection by western blot using the Sha31 antibody. No positivity was detected in any of the experimentally inoculated cows. Brain homogenates from a classical scrapie-infected sheep (Scrapie + C) and a BSE-infected cow (BSE + C) were used as positive controls.

Atypical scrapie-inoculated cows were monitored periodically. None of the animals included in the study showed TSE-related clinical signs. Four brain areas (frontal cortex, thalamus, cerebellum and obex) from the inoculated animals and two uninfected age-matched control cows were tested for the detection of PrP^Sc^. None of the tested areas showed PrP^Sc^ accumulation by immunohistochemistry (not shown) or western blotting (Figure 1). When evaluating brain sections from the same areas stained with hematoxylin–eosin, no spongiform changes were found.

**Figure 2:**
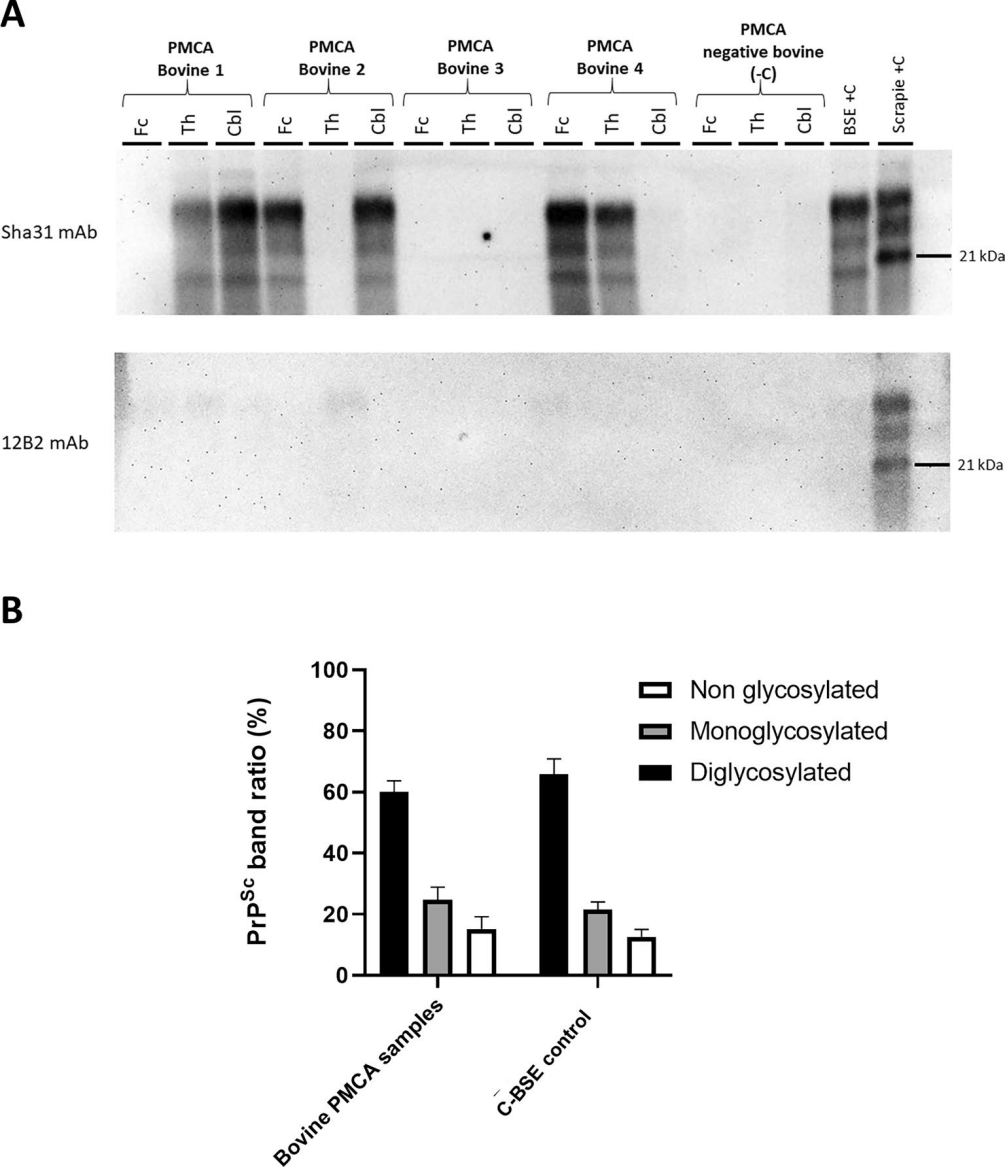
**C‑BSE prions emerge during the in vitro propagation by PMCA of brain isolates from cows inoculated with atypical scrapie.** **A** PMCA reaction products from the frontal cortex (Fc), thalamus (Th) and cerebellum (Cbl) from the four atypical scrapie-challenged cows and a negative control cow were subjected to PrP^Sc^ detection by western blot. Positivity was detected in Th and Cbl samples from Bovine 1; Fc and Cbl samples from Bovine 2; and Fc and Th samples from Bovine 3. No positive reactions were detected in PMCA reaction products from the negative control. PMCA reaction products from a second negative control cow were also subjected to PrP^Sc^ detection by western blot, showing no positive results (results not shown). A classical scrapie isolate (Scrapie + C) and a C-BSE isolate were used as positive controls for western blot. **B** Relative amounts of diglycosylated (black bar), monoglycosylated (grey bar) and unglycosylated (clear bar) glycoforms of PMCA products seeded with brain samples from bovines challenged with atypical scrapie and six C-BSE controls were determined by Image J software. Results are presented as mean ± standard deviation. No significant differences in the relative amount of PrP^Sc^ glycoforms were observed between samples from the study and C-BSE controls.

The previously mentioned brain samples from cows were subjected to PMCA in order to determine whether C-BSE prions could emerge in the atypical scrapie-challenged cows. Brain samples from the negative aged-matched cows were included as a control for the spontaneous generation of prions in vitro. Each brain area was tested in duplicate. After in vitro propagation, positive PMCA amplification was detected in reactions seeded with brain material from 3 out of the 4 atypical scrapie-inoculated cows, in the areas of frontal cortex, thalamus and/or cerebellum. None of the obex samples showed seeding activity (results not shown). No positivity was detected in reactions seeded with the uninfected cows’ brain samples or unseeded PMCA reactions. The glycosylation pattern of the positive PMCA reactions observed in western blot using the Sha31 antibody was characterized by a predominance of the diglycosylated band and a non-glycosylated band at ~19 kDa. No PrP^res^ was detected when using the 12B2 antibody (Figure 2). The observed features are indistinguishable from C-BSE prions and PMCA products from reactions seeded with C-BSE prions. Duplicates of the tested samples revealed identical results when tested by western blot (results not shown).

**Figure 3:**
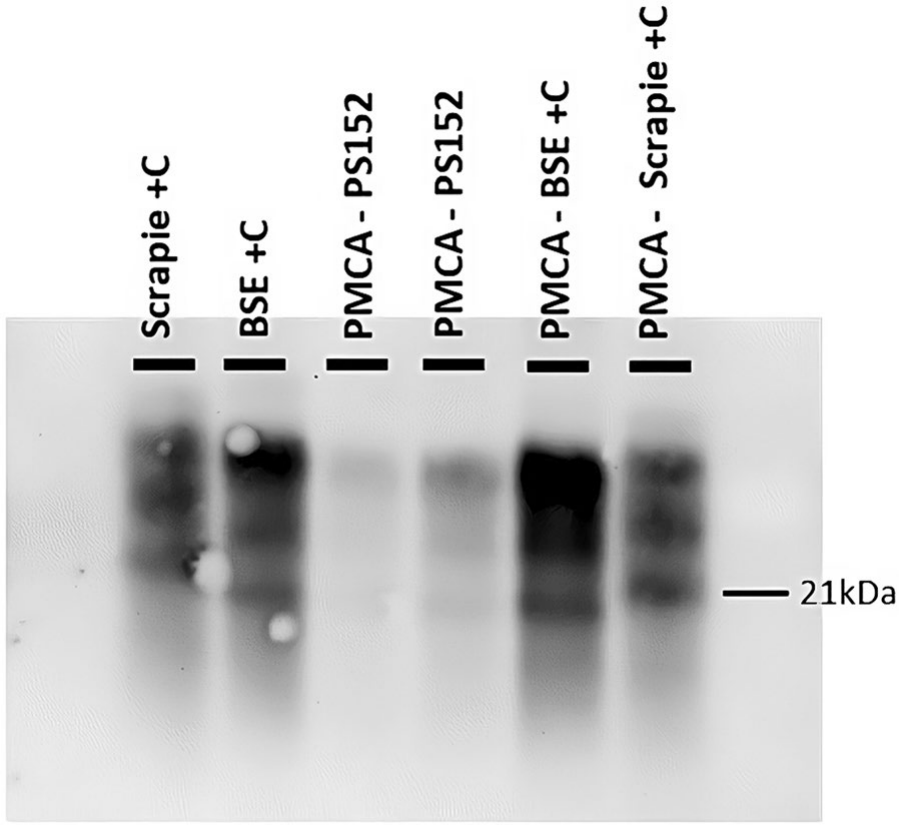
**The atypical scrapie inoculum PS152 shows a C‑BSE signature after propagation in PMCA using TgBov substrate.** PMCA reaction products from the PS152 inoculum (PMCA-PS152) were subjected to PrP^Sc^ detection by western blot. Positive PMCA reactions from cattle C-BSE (PMCA-BSE + C) and scrapie (PMCA-Scrapie C+) propagated in tgBov substrate were used as controls. A classical scrapie isolate (Scrapie + C) and a C-BSE isolate (BSE + C) were used as positive controls for western blot. Using the Sha31 antibody, PS152 PMCA products displayed an indistinguishable glycoform pattern to that of cattle C-BSE and cattle C-BSE propagated by PMCA.

The atypical scrapie isolate PS152 used to inoculate the cows intracerebrally was subjected to PMCA using the previously described conditions (TgBov substrate supplemented with 0.25% final concentration of low molecular weight dextran). The inoculum was diluted at 1/50 and then tested by PMCA in duplicate. After three rounds of PMCA, products were tested by western blot using the Sha31 antibody and amplification was observed. PMCA samples seeded with the original inoculum displayed a C-BSE-like pattern, showing a non-glycosylated band at ~19 kDa and a predominant diglycosylated band (Figure 3).

## Discussion

Previous studies have demonstrated that C-BSE prions can be present as a minor variant in ovine atypical scrapie isolates and that C-BSE can emerge during the passage of these isolates to pigs and bovine PrP mice [7, 8]. These results pointed to atypical scrapie as a possible origin of C-BSE. Therefore, this study was meant to assess the link between atypical scrapie and C-BSE in the natural host of C-BSE, cattle. Although the intracerebral challenge has some limitations and does not reflect the natural transmission process of prions, bioassays using experimental prion inoculation have allowed to identify and describe the transmission mechanisms of these pathogens. Therefore, we decided to challenge cattle with an atypical scrapie isolate.

It is important to note that none of the animals in this study showed any clinical signs of TSE after inoculation with atypical scrapie, according to the results previously obtained in pigs [8]. In addition, the absence of spongiform changes in brain sections, as well as the absence of PrP^Sc^ accumulation by conventional techniques in brain areas from the atypical scrapie-inoculated cows, further highlights the need for highly sensitive techniques such as PMCA to detect low levels of prions. After the in vitro propagation of brain samples from the cows included in this study, seeding activity was detected in reactions seeded with brain material from three out of the four cows, in the areas of frontal cortex, thalamus, and/or cerebellum. Interestingly, none of the samples from the obex, which is one of the most affected areas in prion diseases [14], showed seeding activity. Importantly, the observed glycosylation pattern of the positive PMCA reactions was indistinguishable from that of C-BSE prions and PMCA products from reactions seeded with C-BSE prions. To check whether C-BSE-like prions were present in the original atypical scrapie isolate or if they emerged in the brain of the cows after the inoculation, we performed PMCA of the original inoculum in TgBov substrate, following the same conditions described above. The in vitro amplification of the atypical scrapie inoculum resulted in the propagation of BSE-like seeding activity, biochemically indistinguishable from C-BSE or positive PMCA reactions seeded with brain samples from the inoculated cows, suggesting that, as described before, certain atypical scrapie isolates contain low levels of C-BSE prions [9].

Moreover, in order to rule out a spontaneous in vitro misfolding of bovine PrP during PMCA, we included, as a control for the technique, brain samples from non-inoculated age-matching cows that were also subjected to serial in vitro propagation in TgBov substrate. No positivity was observed in PMCA reactions seeded with samples from these animals, suggesting a true C-BSE-like prion seeding activity and not a spontaneous in vitro misfolding of PrP.

All these results suggest the amplification of C-BSE-like prions during the transmission of ovine atypical scrapie to cows. It is true that, in order to confirm the presence of infectious BSE prions in the challenged cows, strain typing experiments of the PMCA products should be carried out in established mouse lines. Therefore, studies involving a bioassay in bovine and ovine PrP-expressing mice have been started.

Interestingly, the time after inoculation and the BSE-like prion seeding activity were not correlated. As previously stated, the emergence of C-BSE from atypical scrapie has been associated with the presence of low levels of C-BSE prions in the atypical scrapie isolates and our results after the in vitro amplification of the PS152 inoculum support this theory. Therefore, the number of C-BSE conformers contained in the used atypical scrapie isolates may be reduced and not homogeneously distributed, making cows receiving different amounts of C-BSE-like prions. It is true that the emergence of C-BSE-like PMCA seeding activity from the brains of cows could be related to the persistence of prions from the original atypical scrapie inoculum. Previous studies, in which prion seeding activity was detected in the brain of intracerebrally inoculated PrP^0/0^ mice have highlighted the capacity of prions to persist in non-replicative environments [15]. Nevertheless, cows were intracerebrally challenged in the frontal cortex, and seeding activity was detected in caudal regions of their brains but not in more rostral areas such as the frontal cortex. If these positive PMCA reactions were not a bona fide propagation of C-BSE-like prions but associated to inoculum persistence, it would be expected to detect such amplification in the most rostral areas of the brain. Although all these results support a bona fide propagation of C-BSE-like prions, the possibility of PMCA detecting remaining prions of the inoculum, would be definitely ruled out after in vivo bioassays in mouse lines, which are currently being carried out.

The lack of clinical signs of prion disease in cows after inoculation with atypical scrapie contrasts with results from a previous study in which bovine PrP mice (TgBov) were challenged with atypical scrapie isolates and displayed signs of clinical prion disease, developing neuropathological characteristics of C-BSE [7]. In addition, in the mentioned study, after the first passage, signs of clinical prion disease were only observed in a low proportion of the inoculated mice, and several of the inoculated isolates did not lead to PrP^Sc^ accumulation. Three serial passages of atypical scrapie were needed to observe complete attack rates in TgBov mice. Moreover, mice from the first passage that developed clinical signs showed long incubation periods considering the lifespan of a mouse. The cows in this study were also euthanized after a long post-inoculation period (between ~7 and ~11 years). However, the number of C-BSE-like prions present in the original atypical scrapie inoculum was probably too low to produce disease in the cows upon first passage. We also need to consider that TgBov mice overexpress ~8 times bovine PrP^C^, making them more susceptible to develop disease after the inoculation of C-BSE prions.

Further in vivo experiments challenging different mouse lines have been started in order to confirm the infectivity of the PMCA products obtained in this study. However, in conclusion, our findings show that the propagation of atypical scrapie in cattle leads to the emergence of BSE-like seeding activity. This is a concerning issue with far-reaching implications for public health and food safety. The possibility of interspecies transmission of prion diseases and the emergence of new prion strains highlight the critical need for continued surveillance and monitoring of these diseases in both animal and human populations. Early detection of prion diseases is crucial, and highly sensitive detection techniques such as PMCA can play an important role in this regard.
